# Application of Tissue Engineering in Manufacturing Absorbable Membranes to Improve the Osteopromoting Potential of Collagen

**DOI:** 10.3390/bioengineering10010015

**Published:** 2022-12-21

**Authors:** Júlio César Silva de Oliveira, Ana Maira Pereira Baggio, Luan Pier Benetti, Izabela Fornazari Delamura, Edith Umasi Ramos, Vinícius Ferreira Bizelli, Ana Paula Farnezi Bassi

**Affiliations:** Department of Diagnosis and Surgery, School of Dentistry, São Paulo State University (UNESP), Rua José Bonifácio, 1193, Araçatuba 16015-050, SP, Brazil

**Keywords:** biomaterial, regeneration, bone tissue, membrane

## Abstract

The membranes are an important biomaterial that contribute to osteopromotion. This study aimed to evaluate the osteopromotive potential of collagen membranes associated with Hydroxyapatite (HA) in critical size calvaria rat’s defects. Ninety-six Albinus Wistar rats were divided into four groups: (CG) negative control: clot only (CG); positive control: porcine collagen membrane (BG); fish collagen membrane associated with HA (CP); bovine collagen membrane associated with HA (CB), analyzed at 7, 15, 30, and 60 postoperative days. At 30 days, membrane integrity was observed in the CB and fragments in the CP and BG groups were dispersed in the center of the defect. At 60 days, BG demonstrated better results with no statistical difference for the CP group (*p* = 0.199) and a statistically significant difference for the CB group (*p* = 0.013). The inflammatory profiles of the BG and CP groups were similar. Immunohistochemistry demonstrated at 60 days moderate osteopontin staining for the BG and CP groups, light staining for the CB, and intense osteocalcin staining for the BG, while the CB and CP groups demonstrated moderate staining. Microtomography revealed the highest mean bone volume (14.247 mm^3^) in the BG, followed by the CB (11.850 mm^3^), and CP (9.560 mm^3^) group. The collagen membranes associated with HA demonstrated an osteopromotive potential.

## 1. Introduction

The concept of guided bone regeneration (GBR) involves the use of a membrane as a barrier, with or without the use of particulate bone grafts and/or bone substitutes on a defect before primary closure, thus controlling tissue growth [1,2]. Therefore, it is believed that membranes create a protective environment for graft integration by excluding cellular and humoral components that cause bone resorption [3,4].

There are many types of absorbable membranes based on organic polymers, such as collagen and polylactic acid membranes, for example [5,6,7]. The main advantage of absorbable membranes is that a second surgical procedure for their removal is not necessary [8,9,10]. Moreover, biodegradable membranes present fewer complications at the incision line in cases of suture dehiscence, are biocompatible with soft tissue, facilitate hemostasis, and are easy to handle in clinical settings [11,12].

Currently, many membranes are commercially available, all of which differ from each other and try to perform similarly to or better than the market leader Bio-Gide^®^ (Geistlich, Wohlhusen, Switzerland) [13,14]. Thus, the search for high-performance membranes continues to be a reason for research while demonstrating how the membrane is a key component of the GBR process [15,16,17].

During the processing of fish resources, a significant portion is discarded or used to produce low-value fish meal and oil. However, the discarded portion is a rich source of valuable proteins such as collagen, vitamins, minerals, and other bioactive compounds [18,19]. In recent years, the use of fish collagen as an increasingly valuable biomaterial has drawn considerable attention from researchers for its use in biomedical products, owing to its improved physicochemical properties’ stability, mechanical strength, biocompatibility and biodegradability [20,21,22].

In this context, the search for sources that enable collagen extraction has also been conducted. Type I collagen is most abundant in freshwater and marine fish, is extracted from the skin, scales, fins, swim bladder, bones, spine and muscles, and is widely used in tissue engineering [23,24,25,26,27]. Based on these principles, collagen membranes extracted from fish can be used as an alternative for GBR. Another source of type I collagen is the porcine and bovine species, that can be obtained from the dermis, tendon or cortical bone. Currently, there are some biomaterials composed by porcine collagen as Bio-Gide^®^ and composed by bovine collagen associate or not with hydroxyapatite such as Col.Hap-91^®^ (JHS, Biomaterials Sabará, Minas Gerais, Brazil), composed of 25% collagen and 75% hydroxyapatite, which is biocompatible and absorbable [28]. The collagen found in fish, porcine and bovine animals have no difference in its chemical structure. The extraction method can be different as an Acid-based (Acetic acid) or Enzymatic method [29].

Moreover, membranes can provide cell growth by mimicking extracellular matrix, thus allowing growth, proliferation, migration, and differentiation of specific tissues for future implantation [30,31]. The extracellular matrix is composed of the interconnection of protein fibers (collagen and elastin), proteoglycans, and mineral deposits in bone tissue. As a result, some membranes have been associated with hydroxyapatite (HA) nanoparticles with their composition, since their structure is more favorable to osteoblasts, as well as being stable for longer periods [32,33].

The present study aimed to evaluate, through histometric, immunohistochemical and microtomographic analysis, the ostepromotive potential of the collagen membranes associated with HA, using a porcine membrane (Bio-Gide^®^) as positive control, bovine collagen membrane associated with hydroxyapatite (Col.Hap91^®^) and fish collagen associated with hydroxyapatite (CHP^®^ - JHS, Biomaterials Sabará, Minas Gerais, Brazil) as test groups in the repair process of critical bone defects in rat calvaria.

## 2. Materials and Methods

### 2.1. Experimental Design

The Ethics Committee in Animal Experimentation of the Faculty of Dentistry of Araçatuba, UNESP, approved the project prior to the acquisition and accommodation of the animals and before the implementation of any intervention, according to the current standards of the Brazilian College of Animal Experimentation. (Protocol No. 100/2021).

In total, 96 animals (*Rattus novergicus albinus*, Wistar), weighing approximately 300 g, were randomly sent to the vivarium of the Department of Surgery and Diagnosis FOA/UNESP. For this, we used the results of two previous studies [34,35] that ensure an implemented and proven methodology. Throughout the experimental period, they were kept under a cycle of 12 h of light and 12 h of darkness, an ambient temperature of 22 to 25 °C, a ventilation/exhaustion system allowing 20 air changes per hour, relative humidity of 55 ± 5%, plastic codified cages (three animals per cage), and a balanced diet (NUVILAB, Curitiba, PR, Brazil) containing 1.4% Ca and 0.8% P, and ad water libitum.

### 2.2. Experimental Surgery

After 12 h of fasting preoperatively, the animals were sedated with intramuscular administration of Ketamine Hydrochloride (Francotar—Vibrac do Brasil Ltd.a, São Paulo, Brazil) and Xylazine (Rompum—Bayer AS—Saúde Animal, São Paulo, Brazil), at a dosage of 0.7 mL/Kg and 0.3 mL/Kg, respectively. A strict aseptic protocol was adopted, including sterilization of the instruments used, delimitation of the area to be operated with sterile fields, and the use of sterile surgical gowns and gloves. All surgical procedures were performed in the operating room of the vivarium of the Faculty of Dentistry of Araçatuba, UNESP, Brazil.

An initial shaving of the calvaria region was followed by antisepsis using polyvinyl pyrrolidone iodine degermante (PVPI 10%, Riodeine Degermante, Rioquímica, São José do Rio Preto, São Paulo, Brazil), topical PVPI (PVPI 10%, Riodeine, Rioquímica, São José do Rio Black), and apposition of sterile fields.

Soon after the shaving and antisepsis of the animals, a v-shaped incision of approximately 2 cm was made in the occipitofrontal direction, with a total detachment of the flap. Then, with the aid of a 7 mm internal diameter trephine drill coupled at low speed under abundant irrigation with sodium chloride solution, a critical 8 mm diameter surgical defect was created in the central portion of the calvaria involving the sagittal suture, maintaining the integrity of the dura mater.

As proposed, the defects were duly filled only with blood clots in the negative control group, the positive control group were filled with blood clots and the porcine collagen membrane with 0.44 mm thickness (Bio-Gide^®^ Group (BG) *n* = 24) and the test groups, bovine collagen membrane associated with hydroxyapatite (Col.Hap91^®^ Group (CB) *n* = 24), and a fish collagen membrane associated with hydroxyapatite (CHP Group (CP) *n* = 24), placed over the defects, composed by 25% collagen and 75% HA with 2 mm thickness each other.

After the procedure was completed, the soft tissues of the animals were carefully repositioned and sutured in planes using a resorbable thread in the deep plane and monofilament thread with interrupted stitches in the outermost plane.

In the immediate postoperative period, each animal received a single intramuscular dose of 0.2 mL of penicillin G-benzathine (Pentabiotic, Fort Dodge Anima Helf l Ltd., Campinas, SP, Brazil). The animals were kept in individual cages throughout the experiment, with food and ad water libitum, and one person was responsible for the care and distribution of the animals. No animal had postoperative complications.

### 2.3. Histological and Microtomographic Processing

The 7-, 15- and 30-day samples were decalcified, diaphanized, and fixed for histological processing, while the 60-day samples were initially subjected to microtomographic analysis, and only after the acquisitions did, they undergo processing. After making the semi-serial sections at each analysis period (7, 15, 30, and 60 days), some slides were stained with H&E, and others were subjected to immunohistochemistry.

Measurements were performed using an optical microscope (LeicaR DMLB, Heerbrugg, Switzerland) coupled to an image capture camera (LeicaR DC 300F microsystems Ltd., Heerbrugg, Switzerland) and connected to a Pentium III microcomputer with ImageJ Software (version 1.49, National Institutes of Health, Bethesda, MD, USA). The digitized images were recorded in JPEG files for further analysis and projected into a monitor screen.

### 2.4. Histological and Histometric Analysis

The samples were coded such that only one evaluator knew the groups to which they belonged. Moreover, a single examiner performed all analyses.

#### 2.4.1. Neoformed Bone Area

The amount of newly formed bone was quantified from the panoramic reconstruction of the histological sections at 6.3× magnification. Photomicrographs were obtained close to the bone stump and at the center of the defect at 40× magnification at 7, 15, 30 and 60 days for the BG, CP, CB and CG groups. After obtaining the images in the ImageJ software, the values of the area of new bone formation were obtained in one^2^ and summed to obtain the total area.

#### 2.4.2. Inflammatory Profile

The inflammatory profile of the membranes was determined by quantification of inflammatory cells (lymphocytes) and blood vessels. For this, photomicrographs were taken at a magnification of 100×, of which three images were taken per histological section: the first, at the center of the defect; the second, on the right; and the third, on the left. Two histological sections per animal were chosen from samples obtained at experimental times of 7 and 15 days for groups BG, CP and CB, totaling 72 images. After obtaining the images, using the Image J software, a grid containing 130 points was inserted into each image, each cell with lymphocytic (mononuclear) characteristics that touched the point was counted, and the set of points inserted into the same vessel was quantified.

### 2.5. Immunohistochemical Analysis

For immunohistochemical analysis, the prepared slides were subjected to antigenic recovery using phosphate citrate (pH 6.0) and blocked with a solution of hydrogen peroxide diluted in methanol for 15 min. Subsequently, the tissue was blocked with the endogenous biotin obtained from skim milk powder. In the next step, the sections were reacted with specific primary antibodies against osteocalcin (Santa Cruz Biotechnology, Dallas, TX, USA) and osteopontin (Santa Cruz Biotechnology, Dallas, TX, USA). Polyclonal biotinylated secondary goat antibodies produced in donkeys (Jackson ImmunoResearch Laboratories, West Grove, PA, USA) were used with an avidin and biotin amplifier kit (Vector Laboratories, Burlingame, CA, USA). Diaminobenzidine (Dako, Carpinteria, CA, USA) was used as chromogen. In addition, the reaction was terminated by staining the sections with Harris hematoxylin. For each antibody, the immunostaining intensity of the relevant proteins was evaluated semi-quantitatively, assigning different scores according to the number of immunostained cells in the bone repair process. Analysis was performed using an R-DMLB light microscope. Immunostaining intensity was scored from 1 to 4, with 1 indicating no immunostaining and 4 indicating intense staining.

### 2.6. Microtomographic Analysis

The parameters used were as follows: pixel size 11.87 μm, 50 kVp, 0.5 mm aluminum filter, 0.6° rotation, and 180° arc rotation. After scanning, the images obtained were imported into NRecon Reconstruction Software (Skyscan, Bruker, Kontich, Belgium) for three-dimensional (3D) reconstruction of the calvaria in grayscale. After obtaining the 3D images, DataViewer software (Skyscan, Bruker, Kontich, Belgium) was used to determine the volume of interest, which was standardized for all images in the coronal section. The obtained sections were imported into the CT-Analyzer software (version 1.14.4, Skyscan, Bruker, Kontich, Belgium) to evaluate morphometric parameters, such as bone volume (BV), percentage of bone volume (BV/TV), trabecular bone thickness (Tb. th), number of bone trabeculae (Tb.N), trabecular meshwork (Tb. Sp), and percentage of total bone porosity (Po.tot) (Skyscan, Bruker, Kontich, Belgium). The region of interest was delimited according to the rounded morphology of the defects, which was also standardized for all reconstructions (9.74 × 9.74). Soon after, gray tones ranging from 105 to 242 in 40 layers were used. The images were then converted to grayscale to calculate the three-dimensional parameters in millimeters (mm) using the software (CT Analyzer).

### 2.7. Statistical Analysis

The results obtained in the histometric and microtomographic analyses were subjected to the Shapiro-Wilk normality test (*p* > 0.05). For intergroup comparisons, two-way analysis of variance (ANOVA) tests with Tukey’s post-hoc test was used for histometric analyses. For immunohistochemical analysis, qualitative measurements defined by scores from 0 to 3 were performed, and the one-way ANOVA test and post hoc Holm-Sidak test were used for comparison. The significance level was 5%.

## 3. Results

### 3.1. Histological Analysis

At 7 days, the integrity of the membranes and organized vascularized granulation tissue can be observed in the initial phase of the repair process in the CP, CB and BG groups. Cell invasion of the membranes was observed in the CP and CB groups. The GC group showed intense granulation tissue and fibroblast activity. In the second evaluation period, the GC group showed potential osteopromoting activity only in the stumps, while the CP, CB and BG groups still showed membrane integrity. The CP group demonstrated maturation of collagen fibers and intense bone formation towards the center of the defect. The CB group showed a similar behavior associated with the presence of hydroxyapatite fragments within the repair tissue. The BG group showed a significant decrease in cell activity. At 30 days, only the CB group showed membrane integrity. In the CP and BG groups, membrane fragments were dispersed in the center of the defect and bone neoformation was observed in all three groups. The CP and CB groups, however, still showed an intense inflammatory reaction, whereas, in the BG group, the newly formed bone tissue already showed more mature characteristics. No new bone formation was observed in the CG group. In the last experimental period, only the BG group achieved complete closure of the defect in almost all specimens. In the CP and CB groups, a large area of mature neoformed bone, the presence of periosteum and membrane fragments, and hydroxyapatite particles were identified.

### 3.2. Histometric Analysis

**Figure 1:**
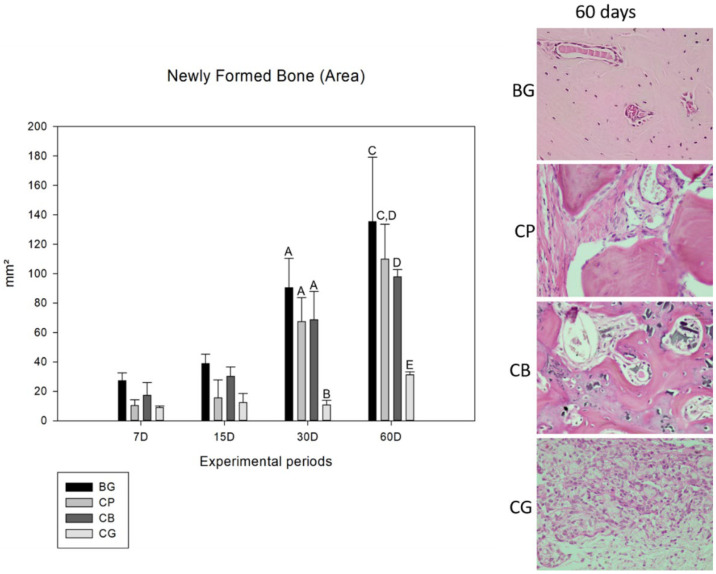
Comparative graph of means and standard deviation of the area of newly formed bone of all groups (BG, CP, CB and CG) for the experimental periods of 7, 15, 30 and 60 days. The capital letters A and B show a statistical difference (*p* < 0.001) between the groups at 30 and C, D, E a statistical difference (*p* < 0.013 for C to D and *p* < 0.001 for C to E and D to E) at 60 days. The 7- and 15-day experimental periods show no statistical difference between the analyzed groups. The photomicrographs at 60 days demonstrate the bone neoformation in the center of the defect for BG, CP and CB groups.

In a comparative analysis of the membrane factor, only the comparison between the CB × CP group (*p* = 0.969) showed no statistical difference, suggesting similar behavior between the groups. The time factor, in turn, did not influence the result, demonstrating that there was no statistical difference only between days 7 and 15 (*p* = 0.504). At 7 days of bone repair, all groups behaved similarly in relation to bone neoformation, with no statistical differences between any of them. At 15 days, despite the greater amount of newly formed bone observed in the samples from the BG and CB groups, no statistical difference was observed between them. At 30 days, in the later period of the bone repair process, the clot group showed the expected results, while the CP group had the most significant increase in the amount of newly formed bone, with no significant difference between the BG (*p* = 0.180) and CB (*p* = 1.000) groups. In the last experimental time analyzed, at 60 days, the BG group had the best performance in the bone neoformation process, followed by the fish collagen membrane, and with no statistically significant difference (*p* = 0.199) between these groups. The CB group had a lower performance than the other membranes, with a significant difference for the BG group (*p* = 0.013), but not for the CP group (*p* = 0.774) (Figure 1).

### 3.3. Inflammatory Profile (Cells and Blood Vessels)

**Figure 2:**
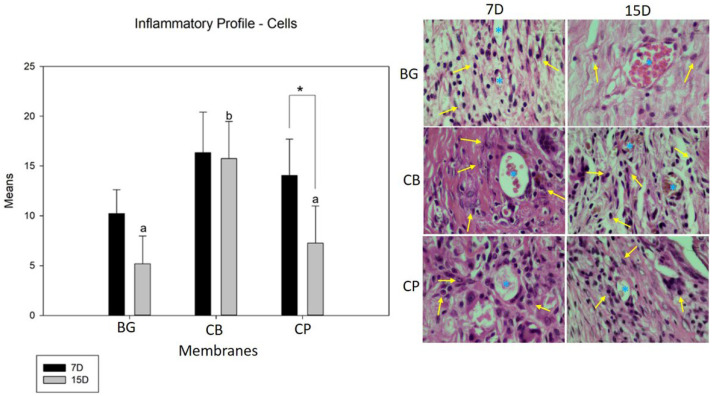
The graph shows the comparisons of the means and standard deviation within and between groups for the analysis of inflammatory cells (lymphocytes) in the period of 7 and 15 days. The lowercase letters represent the statistically significant differences between groups for the period of 15 days (*p* < 0.002), the * demonstrates whether there is a statistically significant difference within the group in the period from 7 to 15 days (*p* = 0.006). In the photomicrographs at 100× magnification, the yellow arrows (↑) indicate the cells (lymphocytes) and the * indicates blood vessels.

According to the comparative evaluation of the relationship between the groups at 7 and 15 days (intra-group), only the fish collagen (CP) group showed a statistically significant difference (*p* = 0.006), while the porcine collagen (BG) (*p* = 0.054) and bovine collagen (CB) groups did not (*p* = 0.789). In terms of cell content, evaluating the relationship between the groups at the end of the seven days showed that the BG group had the lowest number of inflammatory cells, followed by the CP group, but without statistical differences (*p* = 0.658), while the CB group had the highest number of lymphocytes, but with no difference for the GB group (*p* < 0.053). At 15 days, during a similar evaluation, we noticed the same pattern of the inflammatory response, with the CB group displaying a larger number of inflammatory cells, with a statistical difference for the GB (*p* < 0.001) and CP groups (*p* = 0.002). There was no difference in cell content between the CP and BG groups during the 15-day period (Figure 2).

**Figure 3:**
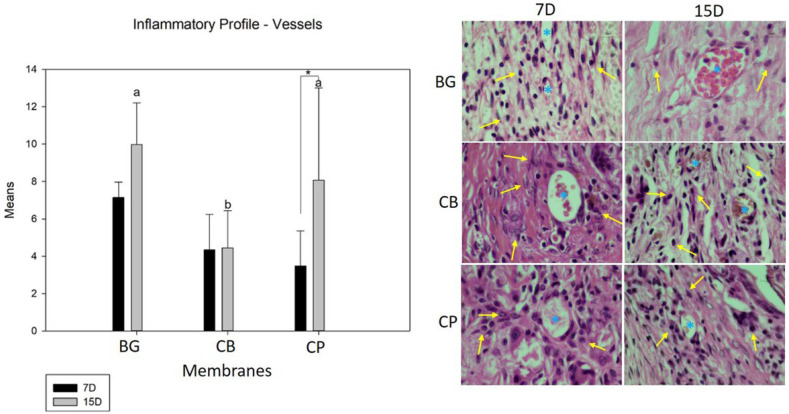
The graph shows the comparisons of means and standard deviations within and between groups for the analysis of the number of blood vessels in the period of 7 and 15 days. The lowercase letters show a statistically significant difference for the period of 15 days (*p* = 0.005); there is no statistical difference for the 7-day period. The * demonstrates whether there is a statistically significant difference within the group in the period from 7 to 15 days (*p* = 0.010). In the photomicrographs at 100× magnification, the yellow arrows (↑) indicate the cells (lymphocytes) and the * indicates blood vessels.

According to the intra-group comparative analysis, only the fish collagen (CP) group showed greater neovascularization from 7 to 15 days (*p* = 0.01) during the bone repair process, which was statistically significant. Moreover, there was no difference in BG group (*p* = 0.096) and CB group (*p* = 0.945). During the 7-day period, there were no significant differences between the groups in terms of osteogenic capacity. In the 15-day period, there was a statistically significant difference in the number of new blood vessels in the BG group (*p* < 0.05) compared to that in the CB group. Although the fish collagen group performed better in terms of forming blood vessels, there was no difference between the CB and CP groups (*p* = 0.073) (Figure 3).

### 3.4. Immunohistochemical Analysis

Immunohistochemical evaluations were performed through a subjective qualitative analysis with attribution of scores, in which immunostaining for osteocalcin and osteopontin, which signal the processes of bone formation and resorption, was characterized.

**Figure 4:**
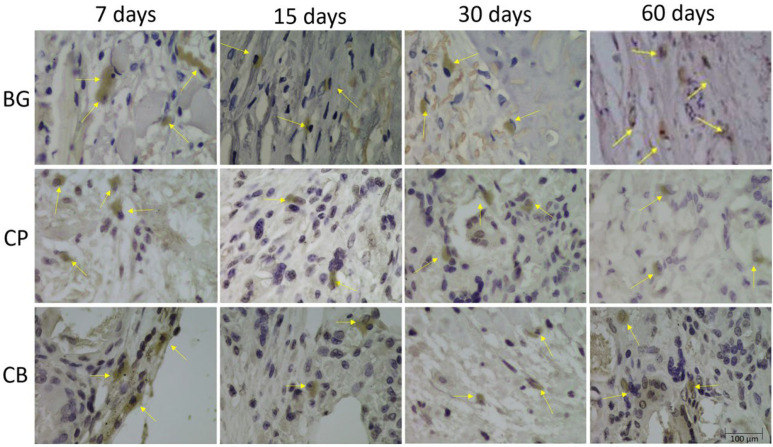
Photomicrographs of groups BG, CP and CB, during periods of bone repair at 100× magnification, showing osteopontin immunostaining (yellow arrows).

At 7 and 15 preoperative days, osteopontin (OP) protein, an important marker that characterizes the early stage of bone tissue mineralization, showed moderate extracellular staining in the BG group, and intense and moderate staining in the CP and CB groups, indicating a possible increase in osteoblastic activity. At 30 days postoperatively, we noticed mild immunostaining in the GB group and moderate immunostaining in CP and CB groups. At 60 days postoperatively, light staining in the CB group, and moderate staining in the BG and CP groups were observed (Figure 4).

**Figure 5:**
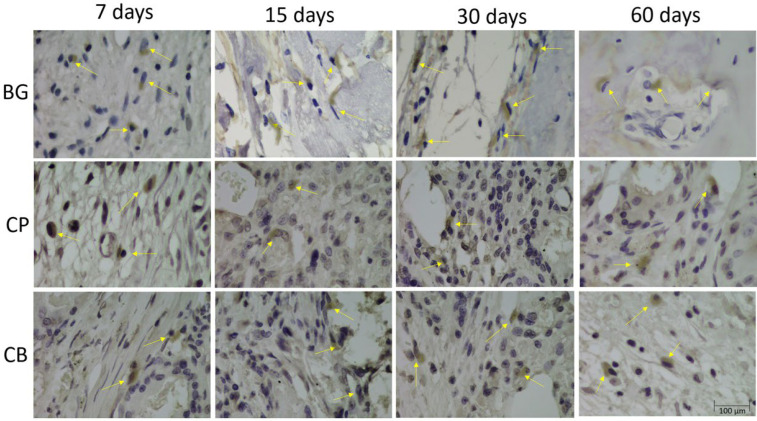
Photomicrographs of groups BG, CP and CB, during the bone repair periods at 100× magnifications, showing osteocalcin immunostaining (yellow arrows).

At 7 and 15 preoperative days, osteocalcin (OC), a protein that characterizes the osteoblastic phenotype, which signals the late phase of mineralization, showed moderate staining in the BG group, moderate staining in the CP group at both times, and light and moderate staining in the CB group. At 30 days postoperatively, we observed intense labeling for osteocalcin in the BG group, intense labeling for osteocalcin in the CP group, and a moderate presence of osteocalcin in the extracellular matrix in the CB group. At the final time of 60 days, we still observed intense staining in the BG group, and moderate staining in the CP and CB groups (Figure 5).

### 3.5. Microtomography Analysis

**Figure 6:**
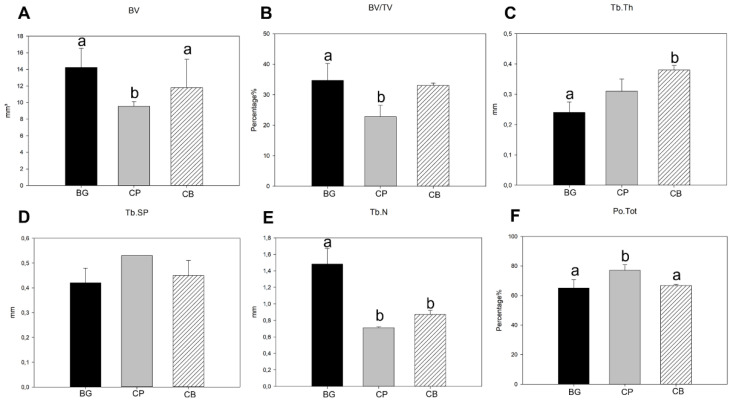
Graphs showing the mean and standard deviation of (**A**) BV, (**B**) BV/TV, (**C**) Tb.th, (**D**) Tb.Sp, (**E**) Tb.n and (**F**) Po. tot in the period of 60 days for groups BG, CP, and CB. Significant statistical differences between groups are represented by different lowercase letters (a, b).

**Figure 7:**
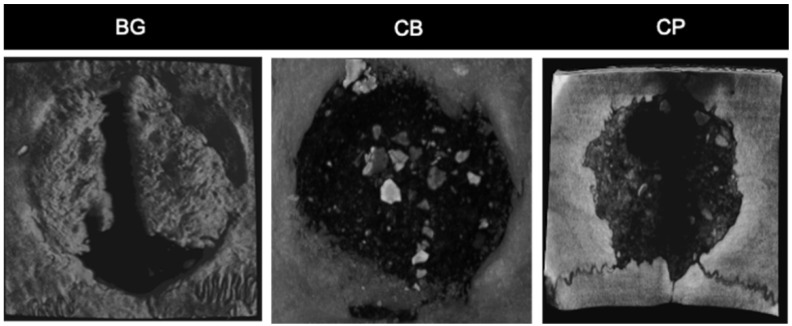
Axial reconstruction of the critical defect in the cap of the animals after 60 days of bone repair, for groups BG, CB and CP. The largest area of bone neoformation is observed in the BG group, followed by the CB group (with the presence of hydroxyapatite granules) and finally the CP group.

For BV/TV, the BG group had the highest means (14.247 mm^3^), followed by the CB group (11.850 mm^3^), and finally the CP group (focus group of our work) (9.560 mm^3^) (Figure 6A), with 34, 7%, 33% and 22.8%, respectively. In the comparative analysis between the groups, the BG group showed a statistically significant difference in the two parameters (BV and BV/TV) in relation to the CP group (*p* = 0.028, *p* < 0.05), but not for the CB group (*p* = 0.625, *p* > 0.05). There was also a difference between the CB and CP groups (*p* = 0.035 and *p* < 0.05) (Figure 6B and Figure 7).

Regarding the trabecular bone, for the average Tb.th, the BG (0.250 mm) and CP (0.310 mm) groups presented very similar results, and the CB group had an average value that was significantly different from the others (0.375 mm), so much so that there was a statistically significant difference between the BG and CB groups (*p* = 0.011) (Figure 6C). Regarding the ratio of Tb.Sp to Tb.N, the GB group again achieved a better result, with smaller spacing in the bone trabeculae (0.427 mm) and the highest number of trabeculae (1.483 per mm), with no statistical difference in Tb.Sp between the evaluated groups (Figure 6D). For the parameter TB.N, the GB group presented a superior response with a statistical difference in relation to the CB and CP groups (*p* < 0.05) (Figure 6E).

The Po.tot evaluation showed the lowest porosity for the BG group (65.130%), followed by the CB group (66.82%), and finally the CP group (77.157%), with a significant statistical difference between the CP group and the BG group (*p* = 0.029) and the CB group (*p* = 0.037), with no difference between the BG and CB groups (*p* > 0.05) (Figure 6F).

## 4. Discussion

Know about the biological behavior of the biomaterials used in clinical practice is essential. The Bio-Gide have a largest literature about its efficiency, between the tested membranes, the CP membranes is not commercially available, and the biological tests are still being conducted to fully understand its biological behavior.

We must observe several factors when using membranes: vascular and cellular permeability, low inflammatory reaction, mechanical resistance and membrane integrity until the neoformed tissue is produced without interference from unwanted tissues in the repair areas [19,35,36].

The use of collagen as a raw material for making a membrane has many advantages, such as the adequate average durability of the barrier effect. As it is absorbed, it allows a single-step surgical procedure to be performed, which reduces patient morbidity and risk for newly regenerated tissues; it has good tissue integration, with less risk of membrane exposure and radiolucency that allows the image of the regenerated bone during healing [31,35,37].

In general, all the membranes evaluated had a good osteopromotion response at the end of 60 days. Compared to the membranes evaluated in this study, porcine collagen membranes demonstrated better bone neoformation and a low inflammatory response across the initial period, as already described by [24,26,35] providing a practically total closure of critical defects with newly formed bone in shorter periods, indicating well-defined and faster biological events.

The fish collagen and bovine collagen membranes proved to be satisfactory in the osteopromotion and osteoinduction processes, presenting a good result in the percentage of bone neoformation, probably because they contain hydroxyapatite, a material with osteoconductive properties. Another important finding was the maintenance of osteoblastic activity at the end times, as observed in the immunohistochemical results, reinforcing the idea of the maintenance of bone formation after the last period evaluated.

The analysis of the inflammatory profile can be influenced by crosslinking, which is a chemical treatment that collagen receives during its manufacture so that more resistant membranes are produced. However, none of the groups demonstrated an intense inflammatory process, suggesting that the biological properties of biocompatibility were maintained during the processing of these membranes. In the groups studied, one of the alternatives to improve the resistance of the membranes, were the addition of HA and several layers to make them thicker. However, when analyzing the thickness and quality of the newly formed bone, thinner bone tissue was found in the CP group, indicating a lower mechanical resistance that did not support tissue pressure, promoting a faster degradation of the membrane, allowing the invasion of epithelial tissue in the defect to be regenerated.

Therefore, it is now understood that the increasingly specific and standardized search for materials causes all the differences in the results of bone reconstruction. Moreover, not only does the type of biomaterial influence the quantity and quality of newly formed bone, but the osteopromoting material plays an equally important role in the choice of osteoconductive/osteoinductive material [19,37].

Although the study demonstrates results that corroborate with other studies and uses a proven methodology, the experimental model has anatomic and physiological limitations already known.

## 5. Conclusions

Using the results obtained in this study, we concluded that all the membranes had the potential to help in the GBR process. The porcine collagen membranes demonstrate a superior biological behavior and still stands out as the better membrane. The fish collagen membrane is superior to the bovine collagen membrane in terms of bone neoformation, with a good potential of neovascularization and decreasing of the number of inflammatory cells. However, the Micro-CT demonstrate that the quality of the neoformed bone at the CP group was slightly better.

## Data Availability

Not applicable.
